# Induction and Consolidation of Calcium-Based Homo- and Heterosynaptic Potentiation and Depression

**DOI:** 10.1371/journal.pone.0161679

**Published:** 2016-08-25

**Authors:** Yinyun Li, Tomas Kulvicius, Christian Tetzlaff

**Affiliations:** 1 III. Institute of Physics – Biophysics, Georg-August-University, 37077 Göttingen, Germany; 2 Bernstein Center for Computational Neuroscience, Georg-August-University, 37077 Göttingen, Germany; 3 Maersk Mc-Kinney Moller Institute, University of Southern Denmark, 5230 Odense, Denmark; 4 Max Planck Institute for Dynamics and Self-Organization, 37077 Göttingen, Germany; 5 Department of Neurobiology, Weizmann Institute of Science, 76100 Rehovot, Israel; 6 School of System Science, Beijing Normal University, 100875 Beijing, China; University of Sydney, AUSTRALIA

## Abstract

The adaptive mechanisms of homo- and heterosynaptic plasticity play an important role in learning and memory. In order to maintain plasticity-induced changes for longer time scales (up to several days), they have to be consolidated by transferring them from a short-lasting early-phase to a long-lasting late-phase state. The underlying processes of this synaptic consolidation are already well-known for homosynaptic plasticity, however, it is not clear whether the same processes also enable the induction and consolidation of heterosynaptic plasticity. In this study, by extending a generic calcium-based plasticity model with the processes of synaptic consolidation, we show in simulations that indeed heterosynaptic plasticity can be induced and, furthermore, consolidated by the same underlying processes as for homosynaptic plasticity. Furthermore, we show that by local diffusion processes the heterosynaptic effect can be restricted to a few synapses neighboring the homosynaptically changed ones. Taken together, this generic model reproduces many experimental results of synaptic tagging and consolidation, provides several predictions for heterosynaptic induction and consolidation, and yields insights into the complex interactions between homo- and heterosynaptic plasticity over a broad variety of time (minutes to days) and spatial scales (several micrometers).

## Introduction

Synaptic plasticity is an important physiological mechanism for learning and memory [1, 2]. The best studied plasticity mechanism is Hebbian or rather homosynaptic plasticity. Homosynaptic plasticity adapts synaptic efficacies by increasing (long-term potentiation; LTP) or decreasing (long-term depression; LTD) them depending on the correlation of pre- and postsynaptic neuronal activities [3, 4]. In contrast, heterosynaptic plasticity adapts synaptic efficacies mainly depending on the postsynaptic activity alone and can effect even presynaptically unstimulated synapses [3, 5–8]. Experiments demonstrated that heterosynaptic plasticity considerably enriches the synaptic dynamics and neuronal functionality and thus the complexity of neural circuits [6, 9]. For instance, the dynamics of heterosynaptic plasticity plays a major role for the allocation of memories [10]. Furthermore, heterosynaptic plasticity prevents homosynaptic induced divergences of the synaptic dynamics and, thereby, stabilizes neuronal circuits [9, 11]. Interestingly, heterosynaptic plasticity occurs in a spatially clustered or localized manner both *in vitro* [12, 13] and *in vivo* [14, 15]. However, the underlying mechanisms are still unknown [16, 17].

Homosynaptic potentiation or depression can be divided into two time scales: (i) early-phase (long-term) plasticity and (ii) late-phase (long-term) plasticity. Early-phase potentiation (ELTP) and depression (ELTD) last for a few hours (up to three hours), while late-phase changes (LLTP and LLTD) last for more than eight hours [18–21]. An early-phase synaptic change can be transferred to a late-phase if two constraints are fulfilled: (i) the changed synapse gets tagged and (ii) a strong activation enables in the postsynaptic neuron the synthesis of plasticity-related proteins which are transmitted back to the tagged synapse. This transfer is named synaptic consolidation and described by the synaptic tagging-and-capture (STC) hypothesis [19, 21–23]. The processes of synaptic consolidation enable several complex dynamics, for instance, cross-tagging. Thereby, a tagged early-phase synaptic change can become late-phase LTP or LTD by protein synthesis triggered by another, unrelated strong stimulus. However, the underlying mechanisms of how *heterosynaptic* changes are transferred from an early-phase to a late-phase are unknown.

To tackle these problems of heterosynaptic plasticity, three questions have to be answered: (i) how is heterosynaptic plasticity induced, (ii) how are these changes transferred from the early- to the late-phase, and (iii) how are these changes restricted to a local subset of synapses.

Several experiments show that the induction of heterosynaptic plasticity (similar to homosynaptic plasticity [24–26]) strongly correlates to the rise of postsynaptic intracellular calcium concentration [11, 27–29]. Different activity-dependent postsynaptic processes, as back propagating action potentials or local dendritic spikes, can induce this intracellular rise. Furthermore, experimental evidences indicate that calcium signals triggered from neighboring spines can be detected within several micrometers [30]. Therefore, we use a calcium-based plasticity model [31], which is already able to reproduce a wide variety of (spike-timing-dependent) homosynaptic plasticity protocols, to induce early-phase homo- and heterosynaptic changes (i). To consolidate the resulting heterosynaptic changes, we assume that the basic mechanisms (synaptic tagging and protein synthesis) are similar to the consolidation of homosynaptic changes. Therefore, we combine the calcium-based plasticity model with a biological reasonable synaptic consolidation model [32] adapted by weight-dependent thresholds for protein synthesis and synaptic tagging (ii). As heterosynaptic changes should be restricted to a local subset of synapses, we introduced an additional term describing the diffusion of calcium from the stimulated to the unstimulated synapse serving as a gating signal of synaptic plasticity (iii). We show that this combined model captures the dynamics of induction and consolidation of homo- *and* heterosynaptic changes as shown in experimental data.

First, we show that the combined model of calcium-based plasticity and synaptic consolidation is able to reproduce experimental results on homosynaptic plasticity [19, 21]. In addition, based on this model, we are able to predict several consolidation paradigms dependent on different stimulation protocols. In the second part, we show that heterosynaptic plasticity can be induced by changes in the postsynaptic calcium concentration and consolidated by the same principles as for homosynaptic plasticity. Here, we found intriguing synaptic dynamics as the induction and consolidation of poorly understood heterosynaptic potentiation. By the assumption of a diffusion process, such heterosynaptic effects are localized and induce a spatial “Mexican hat” shape of plasticity [33] enabling clustered plasticity [16]. Thus, this study provides a further step of understanding how several plasticity mechanisms globally and locally interact with each other to enable the formation of long-lasting changes serving as the basis of memory.

## Materials and Methods

### Basic modeling setup

**Fig 1:**
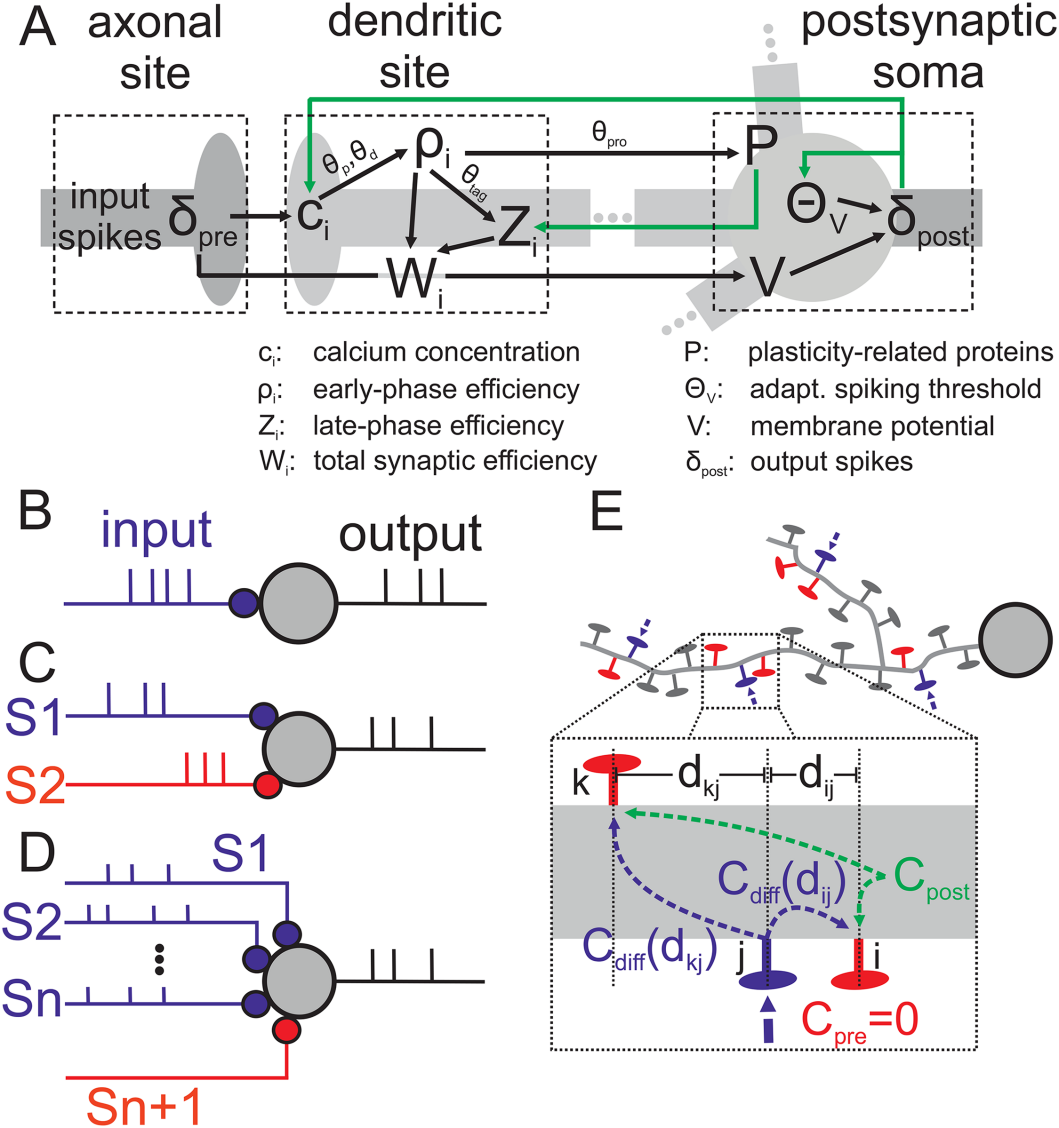
Schematics of setups used throughout this work. (A) Schematic illustration of the interactions between axonal, dendritic and soma-specific quantities used in the model. (B) One neuron receives stimuli from one synapse (small colored circle). We stimulate this synapse by four different protocols (WTET, WLFS, STET, SLFS; for details see main text) to induce different dynamics of synaptic plasticity (viz. ELTP, ELTD, LLTP, LLTD). (C) For cross-tagging and heterosynaptic plasticity experiments, two synapses (*S*1 in red and *S*2 in blue) are connected to one neuron and stimulated independently. (D) Similar to setup (C) but with *n* stimulated synapses (*S*1, *S*2, …, *Sn*) and one non-stimulated synapse (*Sn* + 1). (E) For distance-dependent heterosynaptic plasticity protocols we considered that *n* synapses are stimulated (blue). This leads to the induction of a global postsynaptic calcium signal *C*_*post*_. Synapses (red) near the stimulated ones receive in addition a diffusive calcium signal *C*_*diff*_ which depends on the distance *d* between stimulated (blue) and non-stimulated synapses (red).

In order to investigate how the early-phase of homosynaptic plasticity (ELTP and ELTD) is transformed into the late-phase (LLTP and LLTD), we assume a system consisting of one spiking neuron with one incoming synapse stimulated by a Poisson spike train with given frequency (Fig 1B). Next, to reproduce cross-tagging experiments, comparable to the experimental design [19, 22], we consider one neuron with two incoming synapses (red and blue; Fig 1C) each stimulated by an independent Poisson spike train with different frequency and stimulation pattern. By setting the stimulation of one synapse to zero, we analyze the pure heterosynaptic effect of a stimulated synapse (blue) on an unstimulated synapse (red). This is generalized to *n* stimulated and one unstimulated synapse (Fig 1D). Furthermore, to analyze the spatial restriction of heterosynaptic plasticity, we also considered a setup where synapses are spatially distributed along a dendritic branch with certain inter-synapse distances *d* (Fig 1E).

### Neuron model

For simulating the dynamics of the postsynaptic neuron (Fig 1) we use the biological plausible multi-timescale adaptive threshold (MAT) model [34, 35]. In particular, the MAT model reproduces and predicts precisely neuronal spike timings. Amongst others, such spike timings are a key factor influencing plasticity [31, 36]. The MAT model consists of two parts: i) the membrane potential *V*(*t*) and ii) the spike-history-dependent threshold *θ*_*V*_(*t*). The dynamics of the membrane potential is given by a non-resetting leaky integrator:

$$\begin{array}{c} \frac{dV(t)}{dt}=-\frac{V(t)}{\tau_{V}}+R\sum_{i,j}\delta \left(t-t_{i}^{j}\right)\cdot \left(\rho_{i}+Z_{i}\rho_{0}\right). \end{array}$$ (1)

The part $R\sum_{i,j}\delta \left(t-t_{i}^{j}\right)\left(\rho_{i}+Z_{i}\rho_{0}\right)$ is the sum over all inputs dependent on the neuronal resistance *R*. Synapse *i* contributes to the input current when there is a spike at time point $t_{i}^{j}$. The synaptic input is weighted by two parts; one describes the influences of the early-phase synaptic weight *ρ*_*i*_ and the other the late-phase synaptic weight *Z*_*i*_ scaled with a factor *ρ*_0_. The dynamics of *ρ*_*i*_ and *Z*_*i*_ will be described later.

The dynamics of the adaptive threshold *θ*_*V*_(*t*) is determined by the following equation:

$$\begin{array}{c} \theta_{V}\left(t\right)=\omega +\sum_{k}(\alpha_{1}\;e^{\frac{t-t_{k}}{\tau_{1}}}+\alpha_{2}\;e^{\frac{t-t_{k}}{\tau_{2}}}). \end{array}$$ (2)

The threshold *θ*_*V*_ is composed of a constant *ω* and of multiple time scales dependent on the spiking history of the neuron: *τ*_1_ = 10 ms and *τ*_2_ = 200 ms (*τ*_1_ represents fast transient *Na*^+^ currents and delayed rectifier *K*^+^ currents; *τ*_2_ represents non-inactivating *K*^+^ currents, hyper-polarization-activated cation currents and *Ca*^2+^-dependent *K*^+^ currents [37]). If, at time point *t*_*k*_, the voltage *V*(*t*) is larger than the threshold *θ*_*V*_(*t*), a postsynaptic spike will be elicited and the threshold increases to impede the generation of further spikes. We use the same values for the parameters as in [35], see also Table 1.

**Table 1:** Used parameters if not stated differently elsewhere.

Parameter	value	Parameter	value	Parameter	value
*θ*_*d*_	1.2	*θ*_pro_	0.5 *ρ*_0_	*τ*_1_	0.01s
*θ*_*p*_	3.0	*θ*_tag_	0.2 *ρ*_0_	*τ*_2_	0.2s
*γ*_*d*_	313.1	*κ*	1/6 min	*α*_1_	0.015
*γ*_*p*_	1645.6	*ρ*_0_	0.5 $\frac{\gamma_{p}}{\gamma_{p}+\gamma_{d}}$	*α*_2_	0.003
*c*_pre_	1	*ω*	0.005	*τ*_*ca*_	0.0488s
*c*_post_	0.2758	*τ*_*ρ*_	688.4s	*τ*_*V*_	0.01s
*τ*_*P*_	1 hour	*τ*_*Z*_	6 min	*σ*	9.1844
*γ*	0.1	*R*	0.01		

### Calcium-based early-phase synaptic plasticity

The arrival of presynaptic action potentials and/or the depolarization of the postsynaptic membrane at a synapse induces the entry of postsynaptic calcium through NMDA receptors and voltage-dependent calcium channels [38–42] triggering signaling cascades including protein kinase (LTP) and phosphatase (LTD). These activity-dependent calcium transients are the basic substrate of LTP and LTD induction. The dynamics of the postsynaptic calcium *c*_*i*_(*t*) at synapse *i* is described by the following equation [31]:

$$\begin{array}{c} \frac{dc_{i}\left(t\right)}{dt}=-\frac{c_{i}\left(t\right)}{\tau_{c}}+C_{\text{pre}}\left(t\right)+C_{\text{post}}\left(t\right) \end{array}$$ (3)

$$\begin{array}{c} =-\frac{c_{i}\left(t\right)}{\tau_{c}}+c_{\text{pre}}\sum_{t_{k}}\delta \left(t-t_{k}-D\right)+c_{\text{post}}\sum_{t_{j}}\delta \left(t-t_{j}\right). \end{array}$$ (4)

The parameters *c*_pre_, *c*_post_ are the spike-evoked increases of calcium currents by pre- and postsynaptic spikes at time points *t*_*k*_ and *t*_*j*_, respectively. The time constant *D* = 18.8 ms accounts for the time delay of presynaptic effects on the postsynaptic calcium concentration. We keep the time scale for the calcium dynamics at *τ*_*c*_ = 48.8 ms.

Experimental evidences show that, if the calcium concentration is high, LTP occurs while moderate calcium concentrations induce LTD [38–42]. In other words, the calcium concentration determines the (early-phase) change of the synaptic efficacy *ρ*_*i*_(*t*) as given in the following [31]:

$$\begin{array}{c} \tau_{\rho }\frac{d\rho_{i}\left(t\right)}{dt}=0.1\;\left(\rho_{0}-\rho_{i}\left(t\right)\right)+\gamma_{p}\;\left(1-\rho_{i}\left(t\right)\right)\;\Theta \left[c_{i}\left(t\right)-\theta_{p}\right]-\gamma_{d}\;\rho_{i}\left(t\right)\;\Theta \left[c_{i}\left(t\right)-\theta_{d}\right]+\xi \left(t\right). \end{array}$$ (5)

The early-phase synaptic strength *ρ*_*i*_(*t*) and postsynaptic calcium concentration *c*_*i*_(*t*) are dimension free. If the postsynaptic calcium concentration is *θ*_*d*_ < *c*_*i*_(*t*) < *θ*_*p*_, then *ρ*_*i*_(*t*) decreases by *γ*_*d*_ ⋅ *ρ*_*i*_(*t*) (ELTD); if the calcium concentration is *c*_*i*_(*t*) > *θ*_*p*_, then *ρ*_*i*_(*t*) will increase by *γ*_*p*_ − (*γ*_*p*_ + *γ*_*d*_) ⋅ *ρ*_*i*_(*t*) (ELTP); if the calcium concentration is lower than the threshold *θ*_*d*_, *ρ*_*i*_(*t*) will slowly converge to its initial value *ρ*_*i*_(0) = *ρ*_0_ with the time scale *τ*_*ρ*_/0.1 = 6880 seconds or approximately 1.9 hours [19]. The last term is activity-dependent noise $\xi \left(t\right)=\sigma \sqrt{\tau_{\rho }}\sqrt{\Theta \left[c_{i}\left(t\right)-\theta_{d}\right]+\Theta \left[c_{i}\left(t\right)-\theta_{p}\right]}\eta \left(t\right)$ with *η*(*t*) being Gaussian white noise with unit variance. The Θ- or Heaviside-function is defined as following: Θ[*x*] = 1, if *x* ≥ 0 and Θ[*x*] = 0, if *x* < 0.

Note, the first term is modified compared to the original model [31] to imply only one attractor (the initial value *ρ*_0_). This modification assures that, similar to experiments, potentiated and depressed synapses will return to the initial weight value within the time scale of one to three hours [19, 22].

We use $\rho_{0}=0.5\;\frac{\gamma_{p}}{\gamma_{p}+\gamma_{d}}$ with $\frac{\gamma_{p}}{\gamma_{p}+\gamma_{d}}$ being the saturation value by maximally potentiating the synapse (thus, the maximal possible synaptic weight). We define $\Delta \rho_{i}=\frac{\rho_{i}\left(t\right)}{\rho_{0}}$ as the change of synaptic strength, so that Δ*ρ*_*i*_ > 1 means potentiation and Δ*ρ*_*i*_ < 1 indicates depression.

### Protein synthesis, tagging, and late-phase plasticity

Experimental evidences [19] suggest that synaptic consolidation depends on two thresholds. In the following, we will briefly recapitulate these experiments. Consider two independent synaptic inputs connecting to the same neuronal population (similar to Fig 1C). A weak tetanus stimulation was applied to synapse *S*1 and the resulting synaptic changes decay after one to two hours. Now, a repeated strong tetanus was applied to the second synapse *S*2 one hour before the weak tetanus to *S*1. Then, the synaptic changes at *S*2 get consolidated without effecting *S*1. Remarkably, with the same stimulation protocol except a slightly stronger stimulation at synapse *S*1 (using a single strong tetanus instead of a weak tetanus), the changes at *S*1 are converted from ELTP to LLTP. These experiments indicate two thresholds [19]: one is the high threshold *θ*_pro_ for triggering protein synthesis (e.g., repeated strong tetanus) and the second one is the lower threshold *θ*_tag_ describing the process of synaptic tagging (slightly stronger stimulation).

Thus, the dynamics for protein synthesis depends on the threshold *θ*_pro_:

$$\begin{array}{c} \frac{dP(t)}{dt}=-\frac{P(t)}{\tau_{P}}+\kappa \gamma \;\Theta [(\sum_{i}\left|\rho_{i}\left(t\right)-\rho_{0}\right|)-\theta_{\text{pro}}]. \end{array}$$ (6)

*P*(*t*) represents the number of synthesized proteins, i.e., if the absolute value of the synaptic weight change is larger than *θ*_pro_, *P*(*t*) will increase. Thereby, *κ* dictates how fast the proteins are synthesized which could also depend on the dopamine level [32, 43]. If the sum of synaptic changes is smaller than the threshold *θ*_pro_, *P*(*t*) decays with the time scale *τ*_*P*_ to zero.

The protein dynamics *P*(*t*) directly influences the dynamics of the late-phase synaptic weight *Z*_*i*_(*t*):

$$\begin{array}{ccc} \tau_{Z}\frac{dZ_{i}\left(t\right)}{dt} & = & \gamma \;P\left(t\right)\;\left(1-Z_{i}\left(t\right)\right)\;\Theta [(\rho_{i}\left(t\right)-\rho_{0})-\theta_{\text{tag}}]-\\ & & -\gamma \;P\left(t\right)\;\left(Z_{i}+0.5\right)\;\Theta [(\rho_{0}-\rho_{i}\left(t\right))-\theta_{\text{tag}}]. \end{array}$$ (7)

*Z*_*i*_(*t*) represents a complementary synaptic weight for each synapse, i.e., that the total synaptic weight is determined by *W*_*i*_(*t*) = *ρ*_*i*_(*t*) + *Z*_*i*_(*t*)*ρ*_0_. We have included a separate dynamics for *Z*_*i*_(*t*) for the synaptic consolidation of LTP and LTD: if the synapse with ELTP grows strong enough to get tagged (*ρ*_*i*_(*t*) − *ρ*_0_ > *θ*_tag_), the first term of Eq (7) is non-zero and leads to long-lasting potentiation; vice versa, if the synapse with ELTD is depressed enough to get tagged (*ρ*_0_ − *ρ*_*i*_(*t*) > *θ*_tag_), the second term of Eq (7) becomes non-zero and induces late-phase LTD. Note, both processes, the consolidation of LTP and LTD, require proteins *P*(*t*) to participate (thus, *P*(*t*) ≠ 0) otherwise *Z*_*i*_(*t*) will stop changing. As can be seen from the equation, the termination for *Z*_*i*_(*t*) has three reasons: i) there are no proteins synthesized *P*(*t*) = 0; ii) *Z*_*i*_(*t*) reaches a saturating value: *Z*_*i*_(*t*) = 1 for LTP and *Z*_*i*_(*t*) = −0.5 for LTD; iii) the synapse did not get tagged, i.e., |*ρ*_*i*_(*t*) − *ρ*_0_| < *θ*_tag_. Note, for the reasons discussed later, different to other models [32, 44], here we assume that the induction of protein synthesis and synaptic tagging depends directly on the relation of the early-phase synaptic weight to the thresholds *θ*_tag_ and *θ*_pro_, thus, the combined model has weight-dependent thresholds. Even though the molecular machinery requires different proteins for tagging LLTP and LLTD [22], for simplicity, we assume that the thresholds *θ*_tag_ and *θ*_pro_ for LTP and LTD are the same.

For a better overview, all relationships between the variables of the model are summarized in Fig 1A.

### Distance-dependency of heterosynaptic plasticity

In the postsynaptic neuron several processes (e.g., backpropagating action potentials (bAPs), calcium spikes) lead to the influx of calcium into the dendrite or the dendritic spines (*c*_post_; see Eq (4)). If this calcium signal alone would be sufficient to trigger the induction of heterosynaptic plasticity, many, if not all, synapses on the dendrite would be affected. However, experimental evidence indicates that this heterosynpatic influence is restricted to a local area (several micrometers) surrounding the presynaptically stimulated synapse [30, 45–47]. Thus, we expect that an additional signal is required to trigger heterosynaptic changes. This additional signal can be, besides secondary messengers, calcium diffusing from the externally activated synapse to other sites (Fig 1E). This calcium diffusion *C*_diff_(*d*_*ij*_) is locally restricted (several micrometers; [48, 49]) and depends on the distance *d*_*ij*_ between source (*j*; blue) and target synapse (*i*; red). For simplicity, we assume that synapse *i* is not stimulated (*C*_pre_ = *c*_pre_∑_*t*_*k*__ *δ*(*t* − *t*_*k*_ − *D*) = 0) and only synapse *j* receives external stimulation and is close enough such that calcium can diffuse to synapse *i* (*C*_diff_(*d*_*ij*_) > 0). Interestingly, experimental evidence [50, 51] show that such local calcium currents nonlinearly amplify simultaneous global calcium currents (*C*_post_ = *c*_post_∑_*t*_*ν*__ *δ*(*t* − *t*_*ν*_) with postsynaptic events at time points *t*_*ν*_). Thus, we assume that the existence of local *C*_diff_ could induce such a nonlinear amplification of coinciding *C*_post_ or rather that *C*_diff_ could serve as a gating signal determining when *C*_post_ triggers heterosynaptic changes. Mathematically such gating operations can be formulated as a multiplication with a Heaviside function Θ[*x*]:

$$\begin{array}{c} \frac{dc_{i}\left(t\right)}{dt}=-\frac{c_{i}\left(t\right)}{\tau_{c}}+c_{\text{post}}\sum_{t_{\nu }}\delta \left(t-t_{\nu }\right)\cdot \Theta [C_{\text{diff}}\left(d_{ij}\right)-\theta_{\text{diff}}] \end{array}$$ (8)

with threshold *θ*_diff_ defining when the diffusive calcium current is strong enough to nonlinearly amplify *C*_post_. As the diffusion is noisy and locally restricted [48, 49, 51], we consider that the probability $P(d_{ij})$, which describes whether enough calcium (*C*_diff_ > *θ*_diff_) diffuses from *j* to *i*, decreases with larger distance *d*_*ij*_ between the synapses. Thus, Θ[*C*_diff_(*d*_*ij*_) − *θ*_diff_] can be replaced by $\Theta [P(d_{i}j)-r]$ with uniform random variable *r* (from the interval [0, 1]) and Gaussian distributed probability $P\left(d_{ij}\right)=\text{exp}[-{\left(d_{ij}/\sigma_{\text{diff}}\right)}^{2}]$ with constant *σ*_diff_:

$$\begin{array}{c} \frac{dc_{i}\left(t\right)}{dt}=-\frac{c_{i}\left(t\right)}{\tau_{c}}+c_{\text{post}}\sum_{t_{\nu }}\delta_{\text{post}}\left(t-t_{\nu }\right)\cdot \Theta [P(d_{ij})-r]. \end{array}$$ (9)

Note, if synapse *i* is presynaptically stimulated, this stimulation leads to *C*_pre_ > 0 and, thus, Eq (9) can be written as follows:

$$\begin{array}{c} \frac{dc_{i}\left(t\right)}{dt}=-\frac{c_{i}\left(t\right)}{\tau_{c}}+c_{\text{pre}}\sum_{t_{k}}\delta \left(t-t_{k}-D\right)+c_{\text{post}}\sum_{t_{\nu }}\delta_{\text{post}}\left(t-t_{\nu }\right)\cdot \Theta [P(d_{ii})-r]. \end{array}$$ (10)

Apparently *d*_*ii*_ = 0 implies that $P=\text{exp}[-{\left(d_{\text{ii}}/\sigma_{\text{diff}}\right)}^{2}]=1$ and, thus, $\Theta [P(d_{ii})-r]=1$ and Eq (9) becomes similar to Eq (4). In other words, the basic underlying dynamics of homo- and heterosynaptic plasticity are assumed to be similar.

If a group of synapses *n* is stimulated simultaneously, the stimulation (*n* = 1, …, 20) is sparse compared to the total number of synapses on a dendritic tree (about 10^4^). Thus, we consider large distances between activated synapses such that, on average, no synapse receives diffusing calcium currents from two distinct sites (schematically shown in Fig 1E). However, the number of active synapses influences the postsynaptic activity, thus, the postsynaptic calcium influx *C*_post_ and, thereby, each synapse has a weak influence on the calcium dynamics and heterosynaptic plasticity at distant synapses.

### Stimulation protocols

It is experimentally well known that synaptic consolidation depends strongly on the used stimulation protocols [19, 22]. In this study, four typical stimulation protocols are used similar to experimental studies [19, 22]:

1. Strong TETanus (STET): three trains of Poisson spikes for 1 sec at 100 Hz with 10 min intertrain interval, typical for the induction of LLTP.
2. Weak TETanus (WTET): a 100 Hz Poisson spike train for 0.1 – 0.3 sec (specified in figure legends), typical for the induction of ELTP.
3. Strong Low Frequency Stimulus (SLFS): 900 bursts each consisting of three Poisson spikes at 20 Hz and a interburst interval of 1 sec, typical for the induction of LLTD.
4. Weak Low Frequency Stimulus (WLFS); 900 Poisson spikes at frequency of 1 Hz, typical for the induction of ELTD.

Further protocols are described in the main text or figure captions. All protocols are repeated, comparable to experimental setups, 10 times and their averages with standard deviation are shown. Simulations are performed on a standard desktop PC using Matlab.

## Results

Heterosynaptic plasticity is an important mechanism for the dynamics of neuronal circuits [6, 9–11]. Thus, to preserve heterosynaptic influences for similar time scales as for homosynaptic changes, heterosynaptic changes have also to be consolidated or rather transferred from an early-phase to a late-phase state. To analyze the underlying processes, we combine a model of calcium-based early-phase plasticity [31] with a model of synaptic consolidation [32] adapted by weight-dependent thresholds (in the following named combined model).

**Fig 2:**
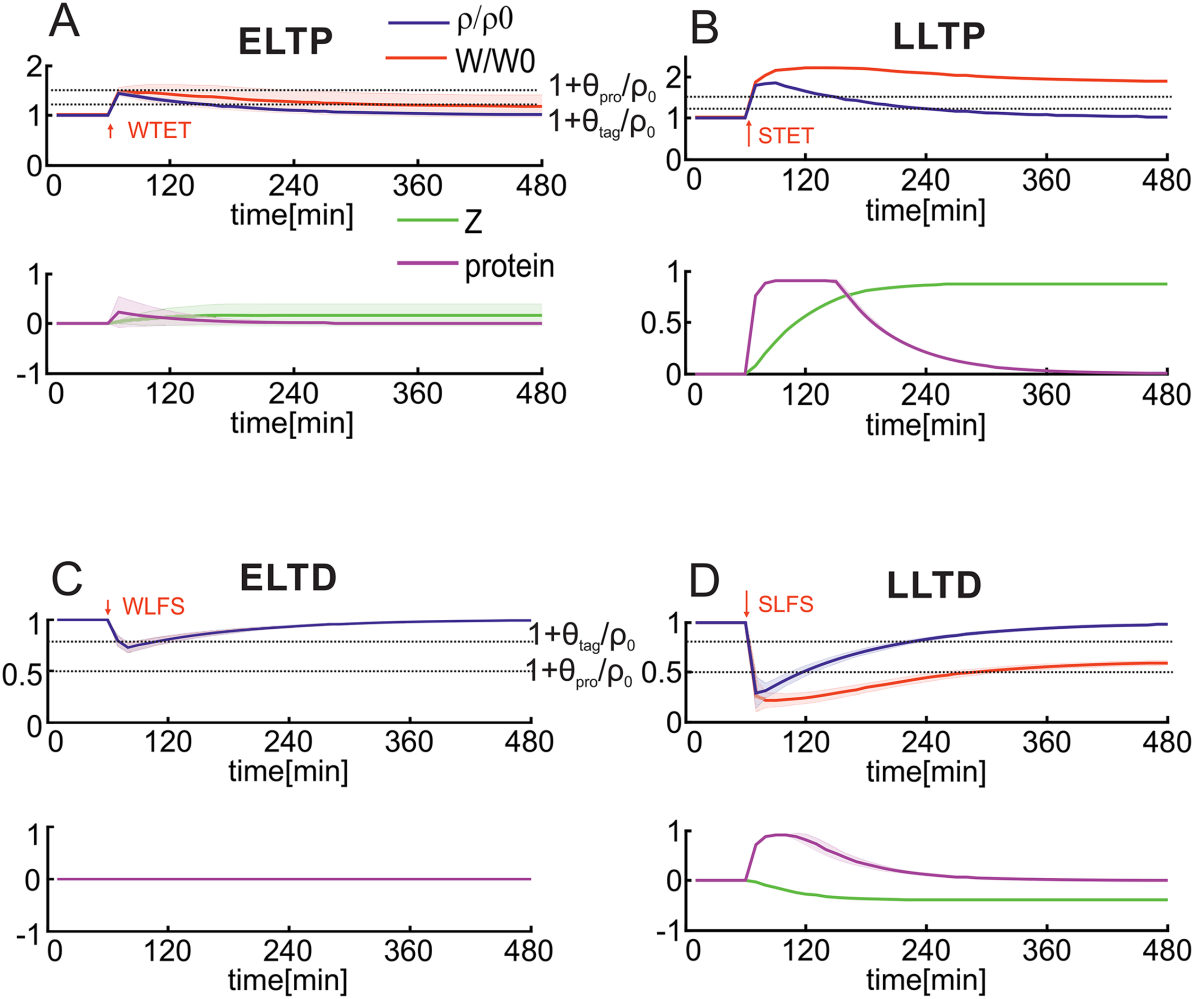
Consolidation of plasticity-induced changes depends on the initiation of protein synthesis. (A) A weak high frequency stimulus (WTET; 0.2 sec) is not able to initiate protein synthesis (purple) and, therefore, only induces ELTP (blue) but not LLTP (green) leading to a fast decay of synaptic changes (red). (B) In contrast, a strong high frequency stimulus (STET) initiates protein synthesis (Δ*ρ* > *θ*_pro_) and, thereby, causes long-lasting changes (LLTP). (C,D) Similar effects arise for the induction of LTD by low frequency stimulation; (C) WLFS leads to ELTD and (D) SLFS leads to LLTD. System Fig 1B is used.

**Fig 3:**
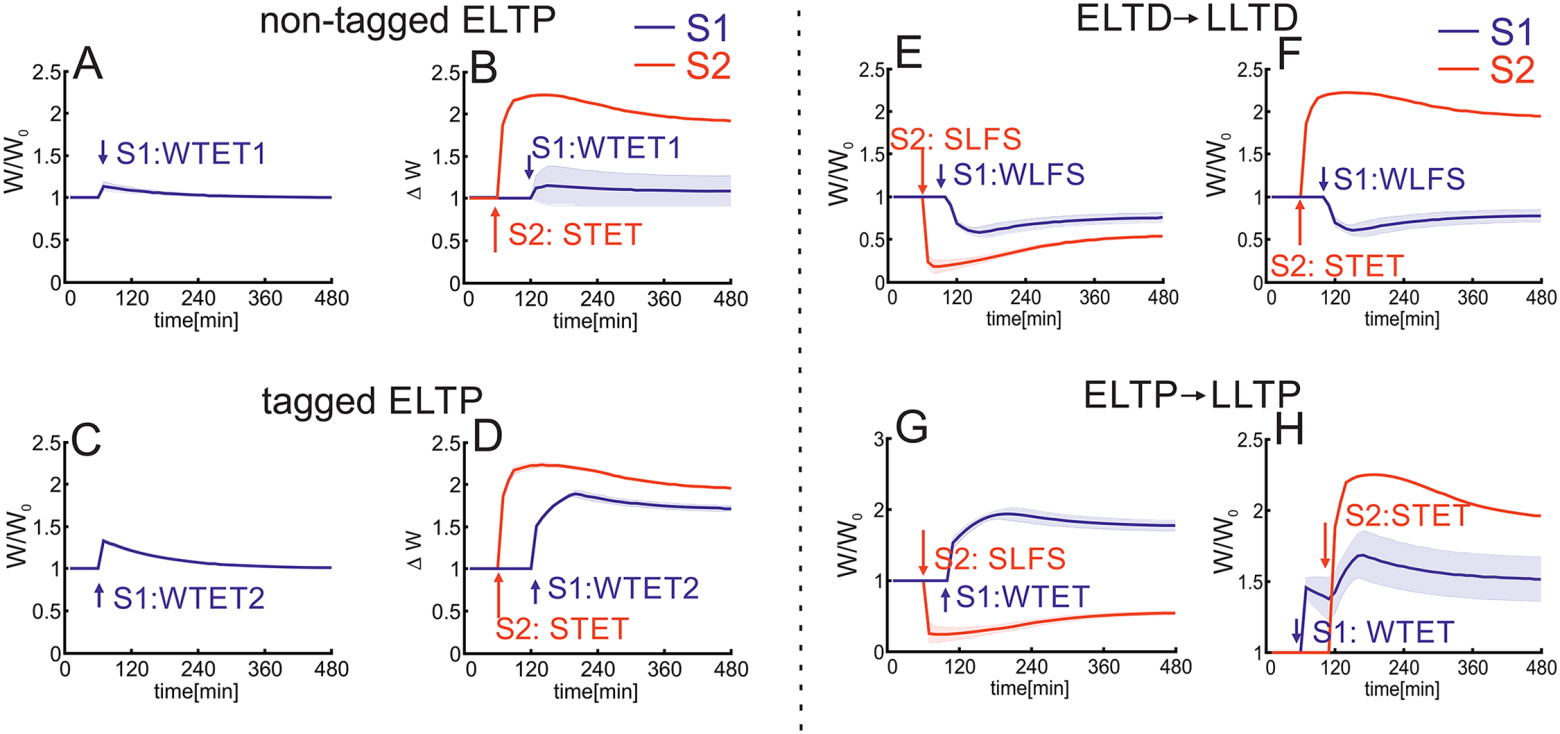
Consolidation of plasticity-induced changes depends on synaptic tagging and cross-tagging. For the consolidation of synaptic changes two requirements have to be fulfilled [19]: postsynaptic protein synthesis (Fig 2) and synaptic tagging. (A,B) A brief (0.1 sec) and weak tetanus stimulation (WTET1) induces ELTP (*S*1; blue). (B) However, despite protein synthesis being initiated by another stimulus (STET at *S*2; red), ELTP at *S*1 cannot be consolidated. (C,D) If the same weak stimulus is provided for a longer duration (e.g., 0.2 sec; WTET2), the resulting early-phase changes are larger. (D) Now, the change reaches the tagging threshold and initiates a synaptic tag, therefore, enabling consolidation or cross-tagging of the synaptic change from ELTP to LLTP given protein synthesis initiated by *S*2. (E-H) As in experiments (Sajikumar et al., 2007), in the model, cross-tagging is independent of the sequence of stimuli. ELTD becomes LLTD supported by (E) a strong depression (SLFS) or (F) potentiation stimulus (STET). (G,H) The same holds for the consolidation of ELTP. Stimulation duration of WTET is 0.2 sec.

**Fig 4:**
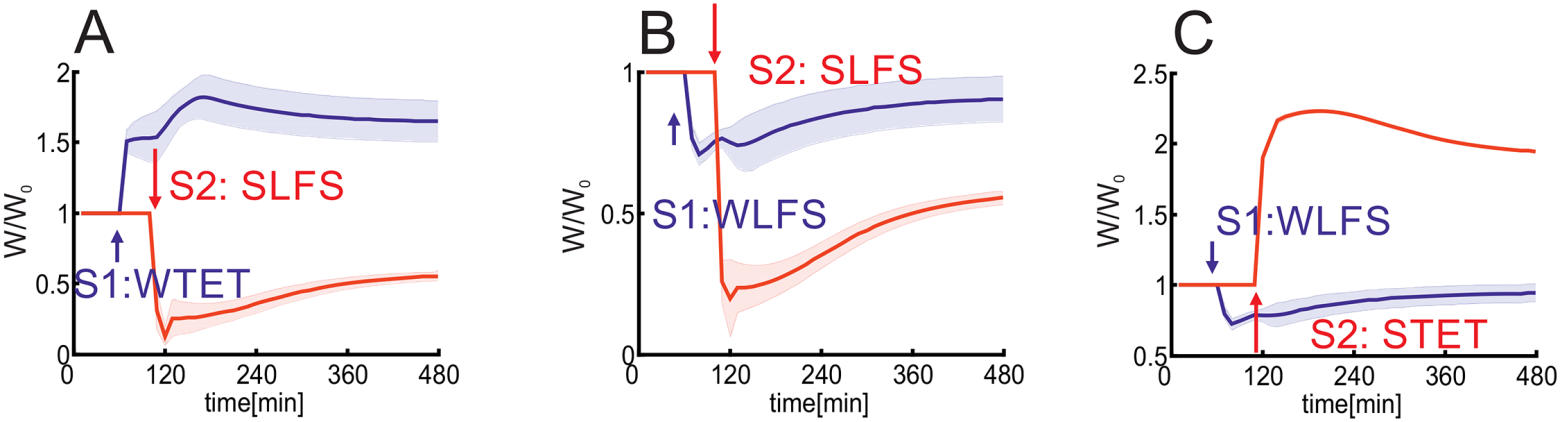
Cross-tagging is not sensitive to the order of stimuli. (A) ELTP (WTET; 0.2 sec) can be consolidated by protein synthesis initiated one hour after ELTP induction (*S*1; blue). Thereby, the strong stimulus at synapse *S*2 (red) can be an LTD- or LTP-induction (Fig 3H) signal. (B,C) The same effect arises for ELTD (WLFS) which can be consolidated supported by a (B) SLFS or (C) STET stimulus.

**Fig 5:**
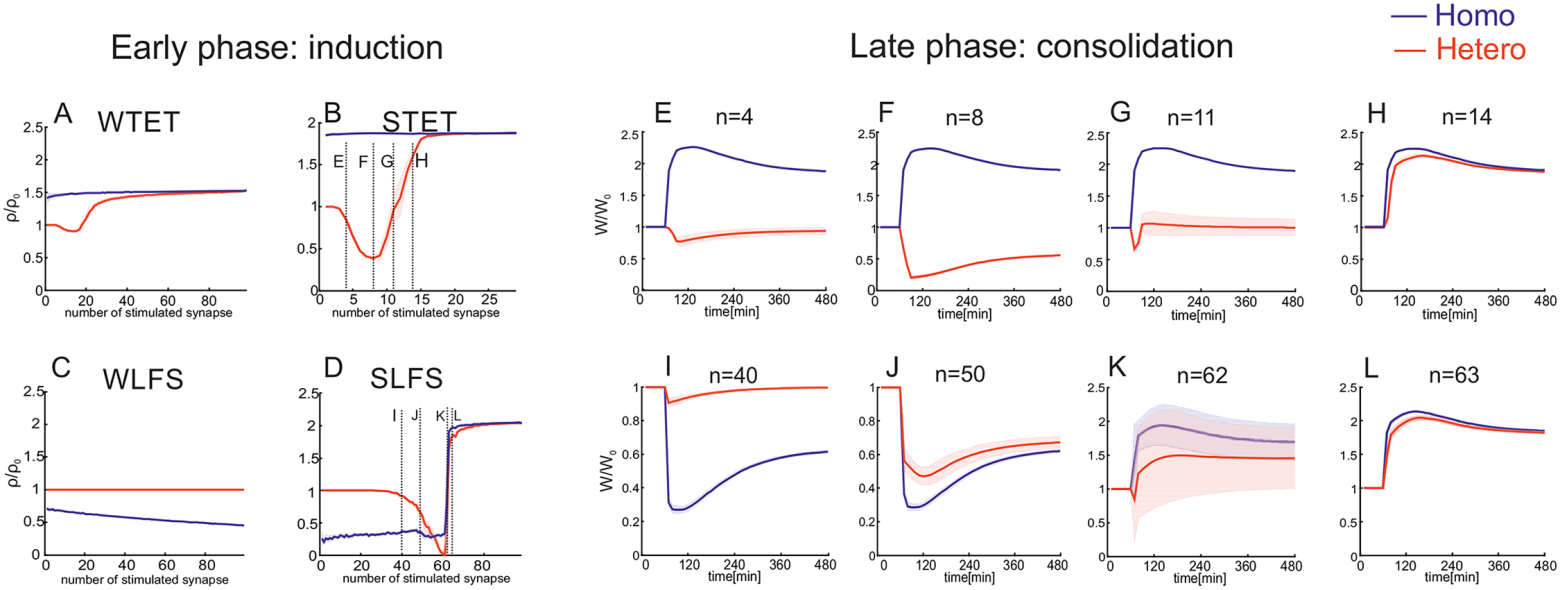
The number of synapses with correlated inputs determines the type and consolidation of heterosynaptic plasticity. (A-D) Varying the number of synapses *n* receiving Poisson spike trains of same frequency and different stimulation protocols enable many types of homo-(blue) and heterosynaptic (red) changes. For each *n*-value the synaptic weight change of Δ*ρ* = *ρ*/*ρ*_0_ (averaged over 10 trials [red and blue] and *n* synapses [blue]). (A) WTET (stimulation duration of 0.3 sec); (B) STET; (C) WLFS; (D) SLFS. (E-H) Applying the same stimulation protocol (here, STET) to a different number of synapses induces different heterosynaptic changes Δ*W*: (E) *n* = 4: ELTD; (F) *n* = 8: LLTD; (G) *n* = 11: ELTP; (H) *n* = 14: LLTP. (I-L) Similar to (E-H) with a SLFS stimulation: (I) *n* = 40: ELTD; (J) *n* = 50: LLTD; (K) *n* = 62: ELTP; (L) *n* = 63: LLTP. System Fig 1E is used.

**Fig 6:**
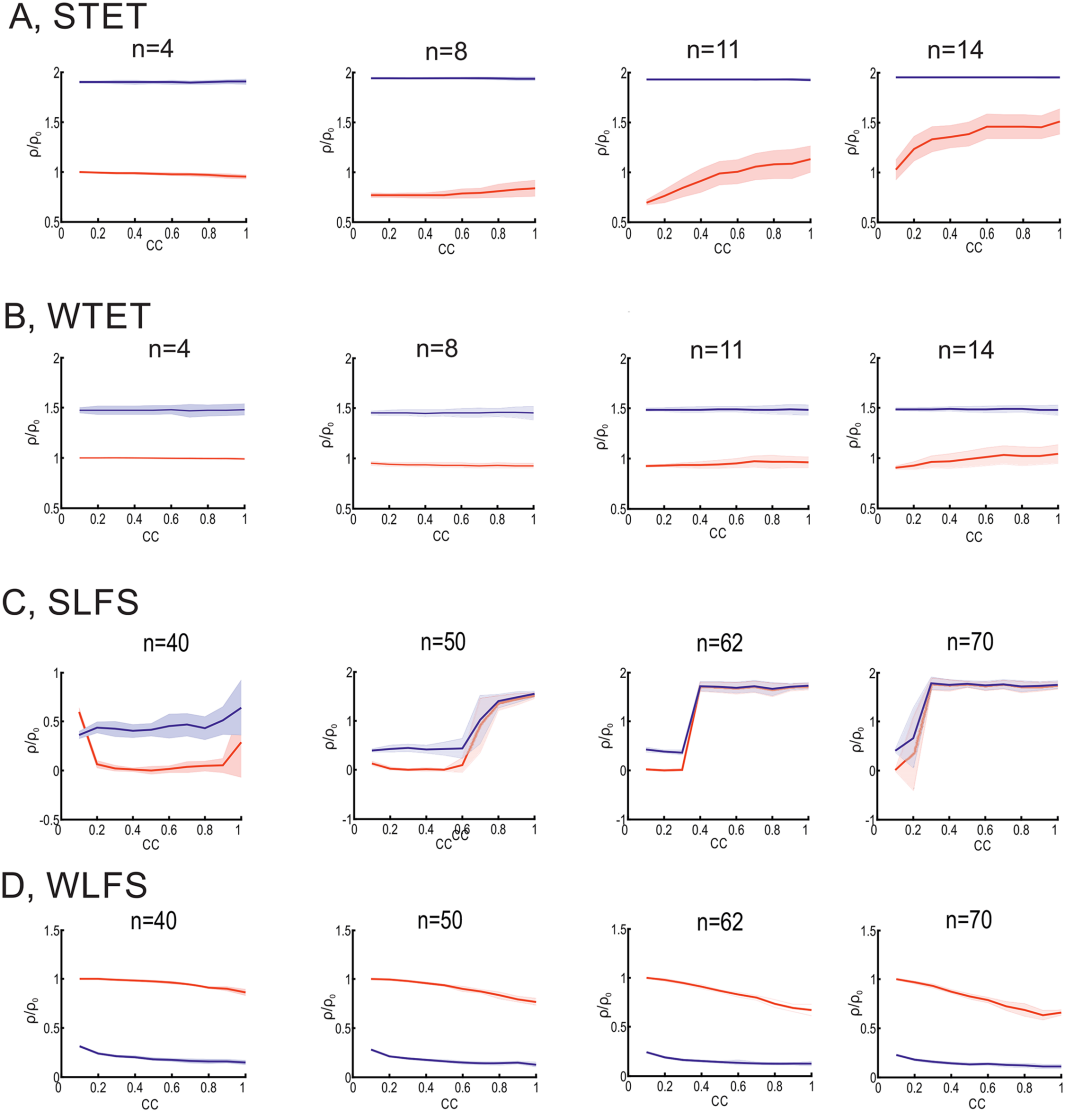
Correlation *CC* of inputs influences the induction of homo- and heterosynaptic plasticity. Averages over 100 trials with standard deviations are shown. In general a higher correlation of input spike trains yields a stronger heterosynaptic effect. (A) STET; (B) WTET; (C) SLFS; (D) WLFS. Details see main text.

**Fig 7:**
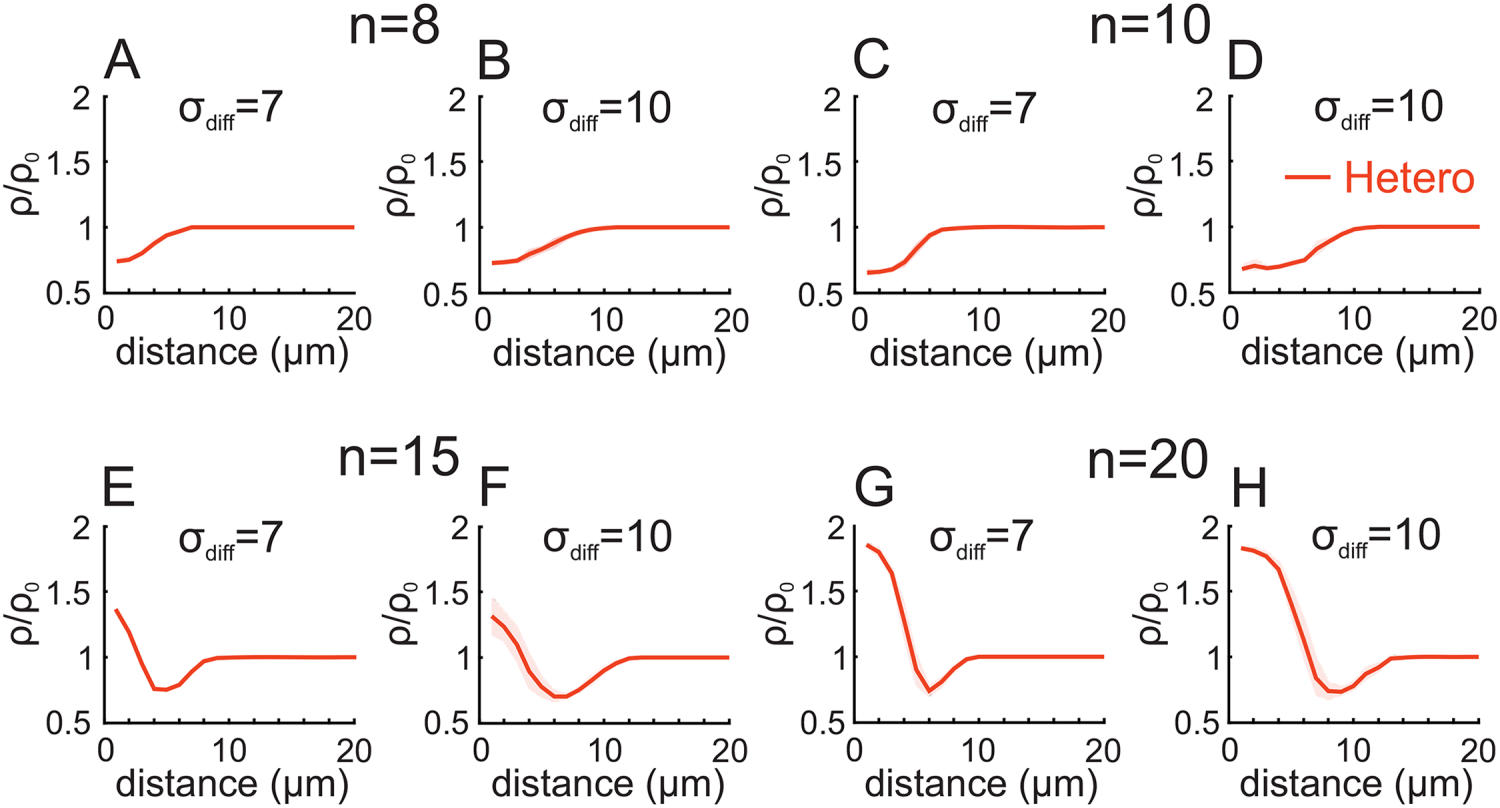
Distance-dependent heterosynaptic plasticity. *n* synapses receive independent Poisson spike trains with 100 Hz for 1 sec yielding a homosynaptic potentiation (not shown here). Due to the diffusion of messengers from the stimulated to unstimulated synapses, the unstimulated synapses (red) are heterosynaptically depressed (A-D) or potentiated and depressed (E-H) depending on their distance to the stimulated synapses. Synapses which are far away from a stimulated synapse (about 10 – 14 *μ*m) are uneffected.

In summary, first, we show that this combined model is able to reproduce generic experimental results [19, 21] regarding the induction of early- and late-phase synaptic changes by the additional assumption of two thresholds each for protein synthesis (Fig 2) and synaptic tagging (Fig 3A–3D). Due to this assumption, cross-tagging dynamics in the model are comparable to experimental observations (Fig 3; [19, 22]). Furthermore, this enables predictions for resulting dynamics using other stimulation protocols (Fig 4). In addition, the combination of the double-threshold mechanism and the calcium-based plasticity model enables the induction of early-phase heterosynaptic changes and their consolidation by transferring them to the late-phase state (Fig 5). Thereby, an important factor influencing the dynamics of homo- and heterosynaptic changes is the correlation of inputs transmitted via groups of synapses (Fig 6). Interestingly, by considering local calcium currents between synapses, the heterosynaptic dynamics can be restricted to a local subset of synapses (Fig 7).

### Consolidation of homosynaptic changes by synaptic tagging, protein synthesis, and cross-tagging

It is well known that different stimulation protocols at the same synapse (Fig 1B) induce different types of synaptic changes as, for instance, early-phase LTP/LTD and late-phase LTP/LTD. For instance, a weak tetanus stimulation (WTET; Fig 2A) induces a change of *ρ* (blue) resulting in a change of the synaptic efficacy *W* (red). Although the early-phase synaptic change Δ*ρ* is above the tagging threshold *θ*_tag_ and, therefore, the synapse is tagged (details can be seen in next section), the change does not become late-phase or consolidated as protein synthesis is not initiated (Δ*ρ* < *θ*_pro_) and *W* decays back to its initial value. The induced plasticity effect decays after a few hours (ELTP). If the stimulation is stronger or longer (STET; Fig 2B), the synaptic change reaches the threshold (Δ*ρ* > *θ*_pro_) and, in turn, triggers protein synthesis *P* (purple) inducing long-term synaptic changes *Z* (green; LLTP). These changes lead to long-lasting alterations of the total synaptic strength *W*. Similar effects arise for low frequency stimulations (WLFS and SLFS) which are used for the induction of LTD (Fig 2C and 2D).

Experimental results indicate that the initiation of postsynaptic protein synthesis is not the only requirement to be fulfilled to consolidate a synaptic change [19]. In addition, the synapse itself has to receive a strong enough presynaptic stimulus so that the synapse will be tagged [19, 23, 32]. Comparable to the initiation of protein synthesis, in the combined model, the linketween the strength of a stimulus and the tagging of the corresponding synapse is given by the early-phase change Δ*ρ*. Consider a neuron with two incoming synapses (Fig 1C), one stimulated by a weak tetanus. Independent of the stimulus duration (WTET1: 0.1 sec; WTET2: 0.2 sec), the WTET induces an early-phase change Δ*ρ* (Fig 3A and 3C) which decays after two or three hours as protein synthesis is not initiated (similar to Fig 2A). Given a strong stimulus (STET) at the second synapse *S*2 (red in Fig 3B and 3D), protein synthesis is initiated (similar to Fig 2B) and synaptic changes at each incoming synapse of this neuron can be consolidated (which happens for *S*2). However, if the weak tetanus at *S*1 (blue) is too short (WTET1), the early-phase change does not reach the tagging threshold (Δ*ρ*_*S*1_ < *θ*_tag_) and, therefore, the synaptic change cannot become LLTP (Fig 3B). In contrast, if the WTET is longer (WTET2), early-phase changes are large enough to pass the tagging threshold (Δ*ρ*_*S*1_ > *θ*_tag_) and the synapse is (cross-)tagged and consolidated by the proteins initiated by the second stimulus (Fig 3D). Thus, by linking the synaptic tagging and protein synthesis with the stimulus strength via the early-phase change Δ*ρ*, the combined model shows similar dynamics as measured in *in vitro* experiments [19].

The assumption of having two thresholds *θ*_tag_ and *θ*_pro_ and the ability of cross-tagging (Fig 3D) enables us to reproduce also other experimental cross-tagging protocols ([22]; Fig 3E–3H). In each protocol a strong stimulus is given to synapse *S*2 (SLFS in Fig 3E and 3G and STET in Fig 3F and 3H) initiating the synthesis of proteins (similar to Fig 2B and 2D). As the protein synthesis is initiated for about three hours (Fig 2B and 2D), tagged synaptic changes induced by a weak stimulation at another synapse (here *S*1), also one hour *after* the strong stimulus, are consolidated (WTET in Fig 3D and 3G and WLFS in Fig 3E and 3F). Thus, early-phase potentiation (depression) can not only be consolidated or cross-tagged by late-phase potentiation (depression) at another synapse (connecting the same neuron) but also by late-phase depression (potentiation).

Note, in Fig 3H the stimulation sequence is reversed: the WTET is provided one hour *before* the strong stimulus. However, due to the fact, that the tag at the synapse stays as long as the early-phase change is above the tagging threshold, the synaptic change can be consolidated. This predicts that the timing could also be reversed for other cross-tagging protocols (Fig 4). For instance, a WTET induced ELTP can be consolidated by a SLFS one hour later (Fig 4A). We expect similar dynamics for early-phase depression (Fig 4 and 4C). Of course, if the timing between weak and strong stimulus is given in a way that either the tag or protein synthesis are not present anymore, the transfer of early-phase changes to late-phase is not possible (data not shown).

In summary, the combined model of calcium-based plasticity and synaptic consolidation with two weight-dependent thresholds for protein synthesis and synaptic tagging shows similar synaptic dynamics as experimental measurements [19, 21, 22]. A synaptic change is transferred from early- to late-phase if a tag is created and the postsynaptic neuron initiates protein synthesis (Figs 2 and 3A–3D; [19]). Thereby, the initiation of protein synthesis can be triggered by the tagged synapse itself (Fig 2) or by another synapse leading to cross-tagging (Fig 3). Whether the synaptic efficacy is depressed or potentiated is not relevant (Fig 3D–3G; [22]) as well as the sequence of tagging and protein synthesis events (Fig 4). However, so far the induced changes are all homosynaptic. In the following we will show the dynamics of heterosynaptic plasticity.

### The induction and consolidation of heterosynaptic plasticity

As shown before, the combined model reproduces experimental results of homosynaptic consolidation and cross-tagging. However, given two synapses connecting to the same postsynaptic neuron (Fig 1C and 1D), they can interfere with each other by the dynamics of this shared neuron. For instance, if synapse *S*1 receives a strong presynaptic stimulus (Fig 1D), then this stimulus can activate the postsynaptic neuron triggering changes in the postsynaptic calcium concentration [31] effecting both synapses inducing heterosynaptic plasticity at the second synapse *S*2. Thereby, it is not essential whether synapse *S*2 also receives presynaptic inputs or is silent.

Thus, the firing rate of the postsynaptic neuron is one important factor determining the induction of heterosynaptic plasticity. Obviously, the postsynaptic activation is influenced by the strength of the incoming stimulus. Amongst others, this input strength can be regulated by the number of stimulated synapses (see S1 and S2 Figs for regulating the resistance instead), as a neuron can receive the stimulus from a population of synchronized presynaptic neurons (e.g., [52]). Thus, a correlated input from a group of neurons can reach a postsynaptic neuron by many synapses enhancing the postsynaptic activity inducing heterosynaptic plasticity. Furthermore, this number of synchronized synapses is variable according to activity-dependent structural changes [53, 54]. Thus, in the following, we test whether the number of stimulated synapses influences the induction and consolidation of heterosynaptic changes.

Consider a postsynaptic neuron with *n* + 1 incoming synapses (Fig 1E) whereby *n* of these synapses receive independent Poisson spike trains of the same frequency and one no stimulation. Thus, the system receives a rate-coded stimulus via *n* inputs. In the following, we will consider again the four protocols WTET, STET, WLFS, and SLFS. Varying the number of synapses enables for these stimulation protocols a rich repertoire of homo- and heterosynaptic changes (Fig 5). A WTET stimulation induces for 3 to 19 input synapses heterosynaptic depression (Fig 5A). However, this change is not tagged and, therefore, does not become consolidated. In contrast, a STET stimulation induces late-phase heterosynaptic depression (Fig 5B, 5E and 5F). For one or two incoming synapses there is no heterosynaptic effect at all (Fig 5B). If 3 ≤ *n* ≤ 5, the stimulus induces an early-phase heterosynaptic depression (ELTD; Fig 5E) comparable to experimental observations [55]. For 6 ≤ *n* ≤ 9 heterosynaptic depression is induced, tagged and, thereby, consolidated by cross-tagging becoming LLTD (Fig 5F). Note, in contrast to a stimulus-dependent synaptic tag [32, 44], only the dependency of the synaptic tag on the early-phase synaptic weight, used in this model, enables the consolidation of heterosynaptic changes. Thus, a group of active presynaptic neurons enables the induction and consolidation of homosynaptic potentiation and heterosynaptic depression which could enable stabilization of dynamics on the network level. However, if the stimulus is transmitted by about *n* = 11 synapses, remarkably, the unstimulated synapse is even potentiated for a short duration (ELTP; Fig 5G). If the number of input synapses exceeds 12, the stimulus induces heterosynaptic plasticity potentiating the unstimulated synapse and, furthermore, inducing a synaptic tag. By this, the unstimulated synapse underwent an heterosynaptic late-phase potentiation (LLTP; Fig 5H). Similar heterosynaptic effects also arise for low frequency stimulation inducing (for low synapse numbers) homosynaptic LTD (Fig 5C, 5D and 5I–5L). Thus, by varying the number of synapses transmitting the stimulus by, for instance, adapting the number of synapses between two neurons (e.g., [54]) or changing the number of neurons encoding the same stimulus (e.g., [56]), the effect of the stimulus on the synapses transmitting the stimulus and on other synapses can be changed accordingly.

Clearly, the heterosynaptic effect depends on the postsynaptic activity enabling the influx of calcium at the unstimulated synapse. The postsynaptic activity, in turn, is influenced by the stimulus frequency of the activated synapses. Besides the frequency (rate-code), however, the correlation [57] between the spike trains affects heterosynaptic plasticity (Fig 6, S3 and S4 Figs). For a high frequency stimulation (STET and WTET in Fig 6A and 6B), correlations in input spiking do not influence significantly the induction of heterosynaptic potentiation. This is due to the fact that the high frequency input is so intensive such that correlations cannot effect the post neuronal firing level. However, for low frequency stimuli (SLFS and WLFS; Fig 6C and 6D), heterosynaptic plasticity is strongly influenced by the correlations in inputs. Especially for the SLFS stimulation protocol at a specific correlation coefficient a transition from heterosynaptic depression to potentiation occurs. With increasing number of correlated input synapses this transition point shifts to weaker correlations. For the WLFS stimulation protocol, although no transition occurs, still the correlation influences the magnitude of heterosynaptic plasticity (depression). Thus, these results imply that synaptic changes are only sensitive to correlations if the average firing rate is relatively low. This can be particularly important considering dynamics in neural networks [57, 58].

However, for all protocols above, the heterosynaptic effect influences all synapses at the postsynaptic dendritic tree. In contrast, experiments show that such effects are limited to only the neighboring synapses of the stimulated one [30, 45–47]. Therefore, we assume that from the stimulated synapse messengers can diffuse to the unstimulated. Amongst others, the increased amount of calcium in the stimulated spine could be such a messenger [48, 49, 51]. If these calcium ions diffuse and the resulting local calcium current coincides with global calcium currents initialized by, for instance, backpropagating action potentials, nonlinear dendritic dynamics lead to additional calcium influx [50, 51] into the unstimulated synapse enabling hetersynaptic plasticity. As diffusion is spatially restricted (by the diffusion parameter *σ*_diff_ based on [48, 49]) and the calcium concentration falls with increasing distance *d* from the stimulated synapse, the nonlinear response and, therefore, heterosynaptic plasticity is also distance-dependent and restricted to a subset of synapses which are close enough to the stimulated ones (Fig 7). This can lead to the fact that, given a homosynaptic potentiation (Fig 7E–7H), nearest-neighbor synapses receive enough diffusive calcium such that they are also potentiated while unstimulated synapses with larger distances receive less calcium and are depressed. As the diffusion is spatially restricted, far away synapses do not receive enough diffusive calcium and the nonlinear increase in calcium influx by coincidence with global signals fail to appear. Thus, these synapses are unaffected and their synaptic strength stays constant. Such a ‘Mexican-hat’-like plasticity profile (Fig 7E–7H) is also found experimentally [33]. Furthermore, given that some of the resulting local synaptic changes (at the nearby synapses) could be tagged while others not, one can get a complex pattern of homo- and heterosynaptic changes over a broad variety of time scales.

In summary, the combined model of calcium-based plasticity and synaptic consolidation with weight-dependent thresholds enables the induction of a rich repertoire of homo- and heterosynaptic plasticity. Thereby, dependent on the stimulation protocol and the number of input synapses, heterosynaptic depression and potentiation can be consolidated in a similar way as homosynaptic changes.

## Discussion

The combination of a generic calcium-based plasticity model [31] with the principles of synaptic tagging and postsynaptic protein synthesis [32] serves as a biological reasonable model of the induction and consolidation of homo- and heterosynaptic plasticity. By introducing weight-dependent thresholds for the initialization of synaptic tags and protein synthesis, this combined model reproduces a wide variety of experimental results [19, 21, 22] as, for instance, the process of cross-tagging. Besides homosynaptic plasticity, the calcium-based model also enables the induction of heterosynaptic changes in both directions: heterosynaptic LTD *and* LTP. By cross-tagging, similar to homosynaptic consolidation, these heterosynaptic changes can be transferred from a short-lived early-phase to a more stable late-phase state.

Heterosynaptic plasticity is an important mechanism to enable several functional properties of neuronal circuits [59]. Several experimental results show that the induction of heterosynaptic as well as homosynaptic plasticity depends on the postsynaptic calcium concentration [24, 27, 28, 33]. For instance, the calcium concentration can be elevated by bursts of back propagating action potentials [1, 60] and further amplified by calcium releases from internal stores [25, 61–66]. Thus, it is straightforward that a calcium-based plasticity model for homosynaptic (spike-timing-dependent) plasticity [26, 31] induces heterosynaptic changes without additional assumptions.

Several theoretical models of activity-dependent synaptic plasticity include homo- and heterosynaptic changes [11, 31, 67–77]. However, heterosynaptic changes are mainly used and discussed as one possibility to induce competition between different synaptic pathways and neurons and they are based on different mechanisms or assumptions as homosynaptic changes [59]. Already the biological questionable assumption of conservation of all synaptic strengths of a postsynaptic neuron (so-called normalization, i.e., ∑_*j*_ *W*_*j*_ = const.) induces heterosynaptic changes, as the homosynaptic strengthening of one synaptic weight leads to the heterosynaptic decrease of all others to keep the sum of weights constant [67, 71, 76–78]. Similarly, the conservation of the postsynaptic neuronal activity also induces heterosynaptic changes. For instance, if one synapse is homosynaptically strengthened by LTP, the postsynaptic neuron receives stronger inputs and, therefore, it will have a higher level of neuronal activity. To counterbalance this increase and return the activity to the previous baseline, the other synapses have to (heterosynaptically) decrease their strengths. This can be achieved by considering activity-dependent homeostasis [56, 72, 75, 79, 80] or a homeostatic sliding threshold defining the activity-conditions of LTP and LTD induction [70, 73, 74, 81]. Although there is biological evidence for such homeostatic processes (reviewed in, e.g., [82, 83]), in general, these processes are much slower than homosynaptic LTP/LTD [84, 85]. Thus, only a few theoretical models include homo- and heterosynaptic plasticity based on the same underlying mechanisms. For instance, the conceptional covariance rule [68, 69] yields homo- and heterosynaptic changes based on the deviations of pre- and postsynaptic activities from their average values. A biologically more detailed model is the here used calcium-based synaptic plasticity rule [31] considering the calcium dynamics as shared underlying mechanism to induce homo- and heterosynaptic changes.

However, experimental data show that the effect of heterosynaptic plasticity is localized to synapses neighboring the stimulated one [30, 45–47]. In our work, the influences of these local processes are simplified to a coincidence detection of global and local calcium signals via, e.g., back propagation of action potentials (global) and diffusion of calcium from stimulated synapses to unstimulated ones within a limited distance (local). This results to a ‘Mexican-hat’-like distance-dependent plasticity (Fig 7) comparable to experimental results [33]. Thereby, calcium, which difuses from the stimulated synapse, serves as an adequate candidate as messenger between synapses [48, 49, 51]. Other diffusive molecules like the strongly local retrograde signaling nitric oxide also influence heterosynaptic plasticity [86]. Note that there is evidence that heterosynaptic plasticity can also be mediated to synapses connecting to other postsynaptic neurons by activity-dependent release of ATP from neurons [87] or adenosine from astrocytes [29]. Interestingly, the consolidation of synaptic changes by plasticity-related proteins could also be locally restricted, as only a fixed number of proteins, the synapses have to compete for, is available and predominantly transported to nearby synapses [88]. These examples indicate that several complex processes influence heterosynaptic plasticity and restrict its induction to a local subset of synapses. Also local processes as the fatigue of internal calcium stores [89] or the adaptation of the nonlinear response of dendrites [13, 90] influence the induction, consolidation and interaction of hetero- and homosynaptic plasticity. However, to analyze the detailed influence of such complex dynamics, the abstract and one-dimensional distance-dependency of the here proposed model has to be extended by, for instance, considering spatial two- or three-dimensional physical simulations.

The mechanism of synaptic-tagging-and-capturing (STC) or synaptic consolidation has several functional advantages for neural systems. For instance, synaptic consolidation prolongs the lifetime of memories stored in neural systems [91] and supports the allocation of a certain memory to a specific subset of synapses [10]. For the induction of synaptic consolidation two requirements have to be fulfilled: the changed synapse has to be tagged and the postsynaptic neuron has to synthesize plasticity-related proteins. These proteins are synthesized in the dendritic compartment or in the nucleus of the postsynaptic neuron [22] and are captured by the local synaptic tags to enable the transfer from an early-phase to a late-phase synaptic change [92]. The biological substrates of synaptic tags can be calcium/camoldulin-dependent kinase II (CaMKII) and/or protein kinase A and protein kinase *Mζ* for LTP-changes and extra-cellular signal-regulated kinase 1/2 for LTD-changes [22, 92]. To combine these STC mechanisms with a calcium-based model, we introduced two weight-dependent thresholds each for the initialization of protein synthesis and the formation of a synaptic tag. This assumption of weight-dependent thresholds enables the consolidation of heterosynaptic plasticity. Different to models with input-dependent tags [32, 44], a synapse does not have to have sufficient presynaptic input to be tagged but instead a sufficient early-phase synaptic change. This implementation of the weight-dependent thresholds into the combined model yields a generic biological reasonable model of the dynamics of synaptic plasticity and consolidation without the need of further assumptions as weight discretization or state-transition probabilities [32, 44, 91]. For instance, models with discrete weight states can only reproduce experimental results about average dynamics of populations of synapses [32, 44, 91] but not the single synapse dynamics as in the current model (Fig 4). Furthermore, in these models, plasticity events resulting in non-tagged synapses do not contribute to the induction of protein synthesis. However, in the combined model even weakly changed but non-tagged synapses can support tagged synapses to get consolidated by initiating protein synthesis. Whether such a contribution of non-tagged synapses exists has to be further investigated.

Remarkably, the dynamic model combined from calcium-based synaptic plasticity, STC principles, and weight-dependent thresholds enables the induction and consolidation of a wide variety of homo- *and* heterosynaptic changes (Fig 5). For instance, the induction of homosynaptic LLTP yields heterosynaptic ELTD or LLTD which is experimentally well studied [5, 33] and a type of competition inducing network stability [59]. Interestingly, besides well-known heterosynaptic LTD, the model shows also the induction and consolidation of heterosynaptic potentiation. To our knowledge, there are only a few experimental studies showing the existence of heterosynaptic LTP [33, 93]. Together with the proposed mechanism of spatial confinement of such heterosynaptic changes, this provides a promising possibility to enable synaptic clustering [16] which, amongst others, self-organizes synapses or dendritic spines according to their input features [51].

The specific combination of homo- and heterosynaptic plasticity depends on the stimulation protocol and the induced input strength. Even for the same input, the induced strength can be adapted by varying the number of stimulated synapses. For instance, the processes of structural plasticity [53, 54, 94, 95] change the number of synapses transmitting a correlated input. Thereby, changing the number of synchronized input synapses induces a wide variety of different homo- and heterosynaptic effects (Fig 5). Interestingly, inducing homo- and heterosynaptic plasticity would alter the neuronal activity which, in turn, adapts, by the slow process of structural plasticity, the number of incoming synapses changing synaptic plasticity. Thus, extending the combined model by the dynamics of structural plasticity could help to understand how processes on different time scales interact with each other resulting in learning and memory on a broad distribution of time scales [84, 96].

In summary, the combination of two biological reasonable models, calcium-based plasticity [31] and synaptic consolidation [32], by weight-dependent thresholds captures physiological findings [19, 21, 22]. In addition, different to other theoretical models (see above), in this combined model heterosynaptic plasticity can be naturally induced by the postsynaptic calcium concentration. Furthermore, with this model at hand we can show that, by the same mechanisms as for homosynaptic plasticity, these changes can be consolidated. This leads to several predictions: for instance, only the consideration of a weight-dependent threshold for initializing a synaptic tag enables the consolidation of heterosynaptic changes (in contrast to an activity-dependent threshold [32, 44] which cannot tag an unstimulated but heterosynaptically changed synapse). Furthermore, we see a clear dependency of the effects of different stimulation protocols on the number of synapses a stimulus is transmitted. This means that, for instance, a stimulus, having a larger representation in a neural network (which is basically encoded by more neurons; [56, 97, 98]) as other stimuli, should not only influence the activity of a postsynaptic (downstream) neuron stronger, but it should also have a different effect on synaptic plasticity on synapses connecting the stimulus-neurons with the postsynaptic neuron and other, neighboring synapses on the dendrite. Thus, the here analyzed transfer between early- and late-phase homo- and heterosynaptic plasticity and their mutual interactions could serve as the basis for the formation, maintenance and interactions of memories.

## Supporting Information

S1 Fig Changing the resistance *R* also influences the induction of homo- and heterosynaptic plasticity. (A) STET; (B) WTET; (C) SLFS; (D) WLFS.
(PDF)

S2 Fig Changing the resistance *R* also influences the induction of homo- and heterosynaptic plasticity dependent on the input correlation. *n* = 100 synapses are stimulated by Poisson spike trains of 100 Hz for 1 sec with different correlations (blue) inducing heterosynaptic changes at the unstimulated synapse (red). **(A)** *R* = 0.001: heterosynaptic plasticity changes from depression to potentiation with increasing correlation; **(B)** *R* = 0.0015: the magnitude of heterosynapitc potentiation increases with increasing correlation in the inputs; **(C)** *R* = 0.0018: heterosynaptic potentiation becomes independent from the correlation; **(D)** *R* = 0.002: heterosynaptic potentiation decreases with increasing correlation; **(E)** *R* = 0.003: heterosynaptic potentiation still decreases with increasing correlation; **(F)** *R* = 0.006: heterosynaptic potentiation becomes again independent from the input correlation.
(PDF)

S3 Fig Changing the correlation between inputs influences Δ*W* and the average postsynaptic neuronal activity (during stimulation). (A) STET; (B) WTET.
(PDF)

S4 Fig Changing the correlation between inputs influences Δ*W* and the average postsynaptic neuronal activity (during stimulation). (A) SLFS; (B) WLFS.
(PDF)
