# Supplementary figures and images for: Induction and Consolidation of Calcium-Based Homo- and Heterosynaptic Potentiation and Depression
Source: PLoS One. 2016 Aug 25;11(8):e0161679. doi: 10.1371/journal.pone.0161679 (PMC4999190; doi:10.1371/journal.pone.0161679)

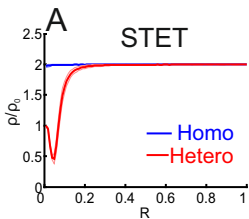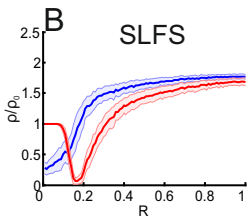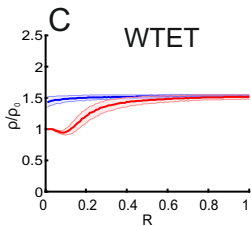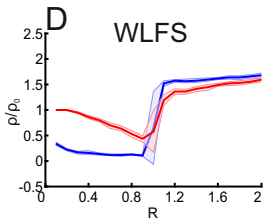

Supplement: S1 Fig — (A) STET; (B) WTET; (C) SLFS; (D) WLFS. (PDF) [file pone.0161679.s001.pdf]

— Homo  
— Hetero

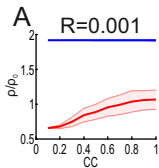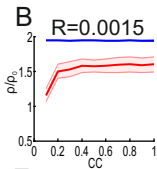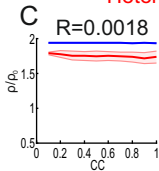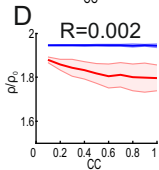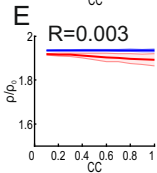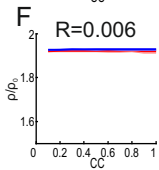

Supplement: S2 Fig — n = 100 synapses are stimulated by Poisson spike trains of 100 Hz for 1 sec with different correlations (blue) inducing heterosynaptic changes at the unstimulated synapse (red). (A) R = 0.001: heterosynaptic plasticity changes from depression to potentiation with increasing correlation; (B) R = 0.0015: the magnitude of heterosynapitc potentiation increases with increasing correlation in the inputs; (C) R = 0.0018: heterosynaptic potentiation becomes independent from the correlation; (D) R = 0.002: heterosynaptic potentiation decreases with increasing correlation; (E) R = 0.003: heterosynaptic potentiation still decreases with increasing correlation; (F) R = 0.006: heterosynaptic potentiation becomes again independent from the input correlation. (PDF) [file pone.0161679.s002.pdf]

# A STET, $n=4, 8, 11, 14$

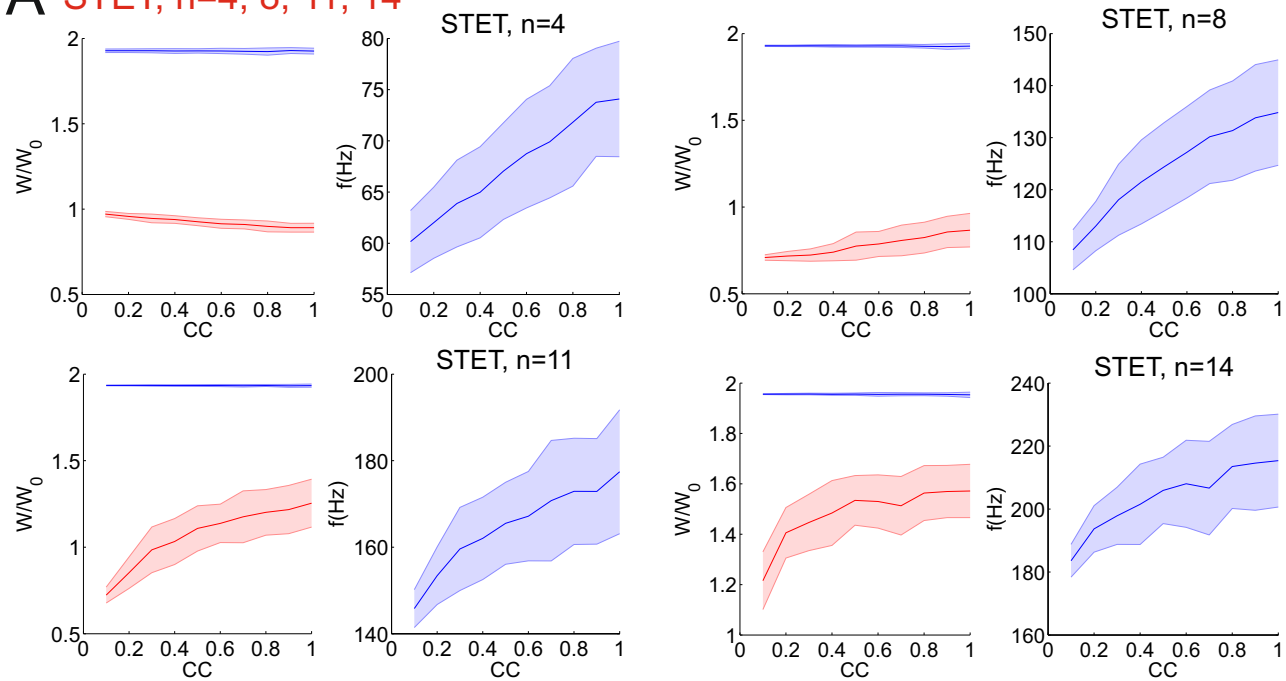

# B WTET, $n=4, 8, 11, 14$

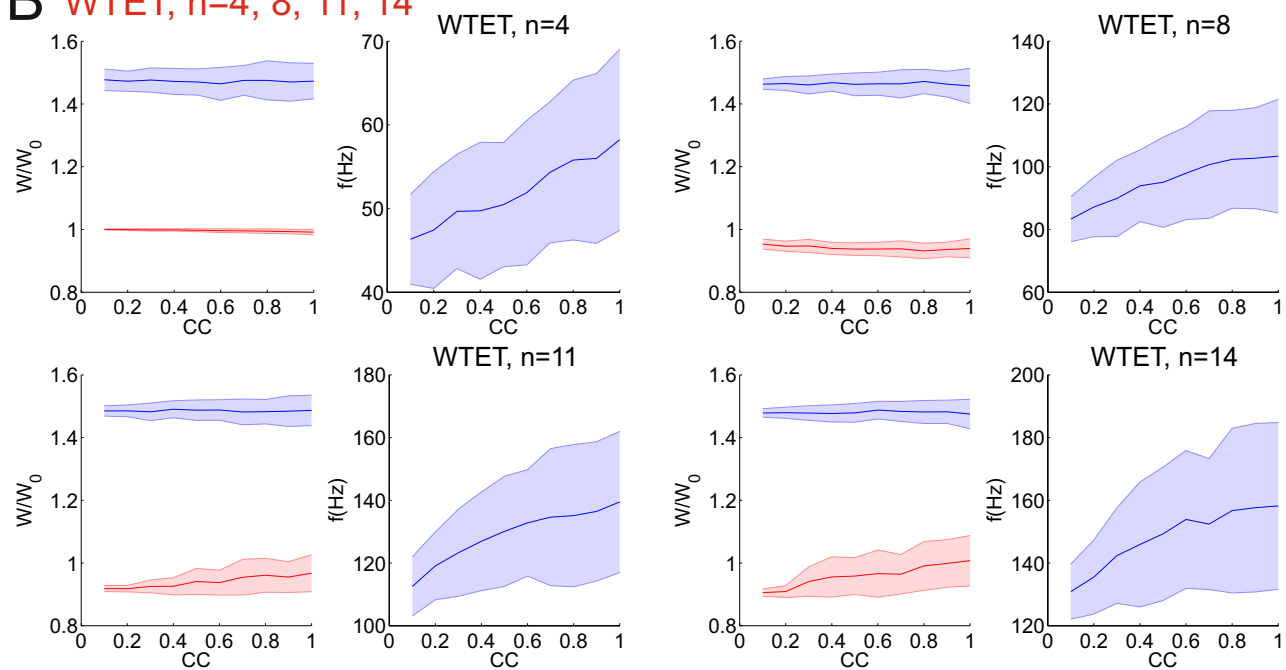

Supplement: S3 Fig — (A) STET; (B) WTET. (PDF) [file pone.0161679.s003.pdf]

# A SLFS, $n=40, 50, 62, 70$

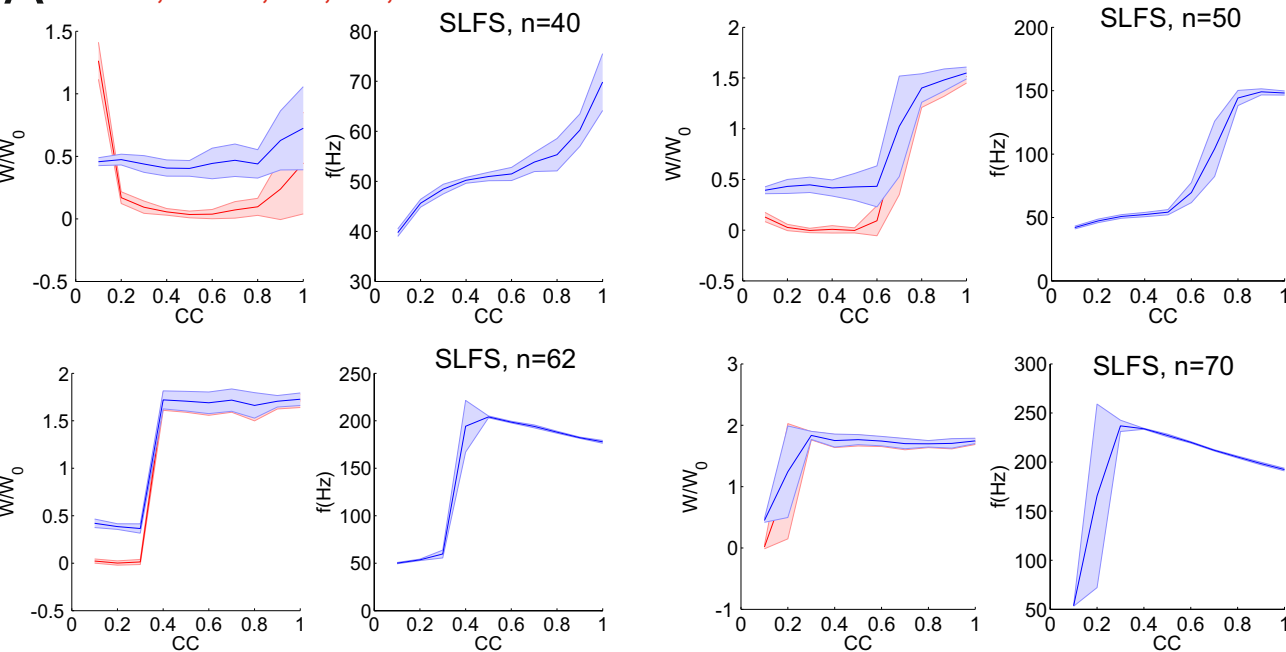

# B WLFS, $n=40, 50, 62, 70$

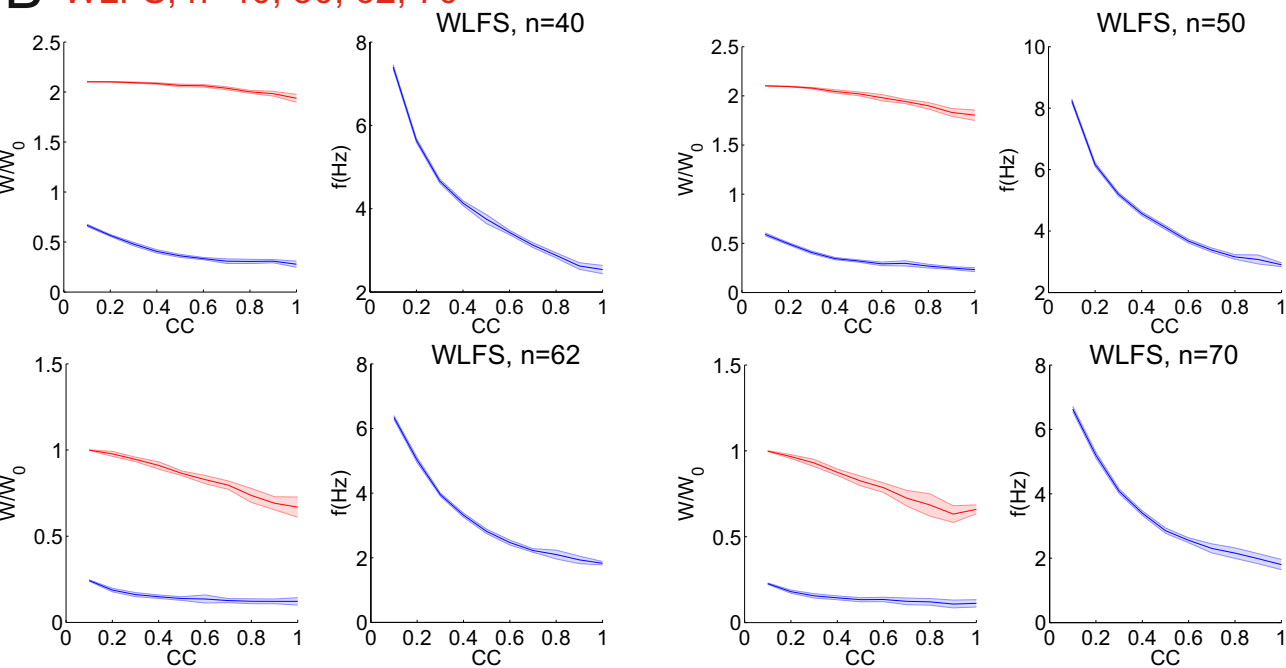

Supplement: S4 Fig — (A) SLFS; (B) WLFS. (PDF) [file pone.0161679.s004.pdf]
